# Addressing Drug Shortages at Mediclinic Parkview Hospital: A ‎Five-Year Study of ‎Challenges, Impact, and Strategies

**DOI:** 10.7759/cureus.76377

**Published:** 2024-12-25

**Authors:** Mohammed Sallam, Albert Oliver, Doaa Allam, Rana Kassem, Mais Damani

**Affiliations:** 1 Department of Pharmacy, Mediclinic Parkview Hospital, Mediclinic Middle East, Dubai, ARE; 2 Department of Management, Mediclinic Parkview Hospital, Mediclinic Middle East, Dubai, ARE; 3 Department of Management, School of Business, International American University, Los Angeles, USA; 4 Department of Clinical Pharmacy, Mediclinic Parkview Hospital, Mediclinic Middle East, Dubai, ARE

**Keywords:** drug shortages, hospital pharmacy, medication management, mediclinic middle east, operational efficiency, pharmaceutical supply chain, policy reforms, risk management, strategies

## Abstract

Background

Drug shortages have become a significant challenge globally, affecting healthcare delivery and patient outcomes. This study aimed to assess drug shortages’ prevalence, causes, and impact at a tertiary care hospital in Dubai, the United Arab Emirates (UAE), providing actionable insights for future mitigation strategies.

Methods

A retrospective descriptive study was conducted at Mediclinic Parkview (MPAR) Hospital, part of Mediclinic Middle East (MCME), UAE. Data were collected from January 2019 to December 2023. Reported drug shortages were analyzed to assess their frequency, duration, causes, and management, with a focus on identifying trends and underlying factors.

Results

Drug shortages peaked at 995 in 2020, particularly during the COVID-19 pandemic. The median time spent managing shortages reached 19.5 days per shortage in Q3 2020. Oral forms accounted for the highest frequency (n = 2231), representing 61% of all shortages, followed by topical forms (n = 414, 11%) and injection forms (n = 386, 10%). Most affected drugs were in the infectious disease (n = 547, 15%), cardiovascular (n = 387, 11%), and respiratory (n = 330, 9%) categories. Drug shortages were driven by regulatory issues and manufacturing delays (39%), unknown reasons (29%), and supply chain disruptions exacerbated by the pandemic (10%). A monopoly environment worsened the situation and limited sourcing flexibility, with 66% of shortages linked to zero supply competitors. Tirzepatide (n = 20) and oseltamivir (n = 18) were the drugs most frequently reported to be unavailable over the 60-month study interval. Regarding management efforts, 80% of the time was spent gathering information and communicating with the different stakeholders. The hospital’s response included contacting prescribers for alternatives and increased reliance on internal procurement and inter-pharmacy coordination. These shortages caused significant operational strain, with increased workloads and higher costs.

Conclusion

The study highlighted the need for adopting proactive measures, improved strategies, enhanced communication, and better preparedness to address future drug shortages. Key actions involved investing in technology, strengthening supplier relationships, and advocating for policy reforms to mitigate risks and ensure continuity of care.

## Introduction

The shortage of medications required to treat medical conditions is a global problem within the healthcare system and affects the entire continuum of care, including all patient settings [1,2]. Indeed, such medications are essential to relieve disease-related symptom burden, minimize the negative impact on the patient’s quality of life, and ensure optimal sickness management and functional capacity [3]. There is an increase in the frequency and complexity of medication unavailability [4,5], with limited academic research and no consensus among stakeholders regarding the causes and safety consequences of drug shortages [6,7]. Therefore, the primary aim of this study is to describe the experience of a tertiary care hospital in the United Arab Emirates (UAE) concerning drug shortages, their prevalence encountered root causes, and their severity, as well as the strategies to mitigate and manage medicine shortages. This study also seeks to identify the characteristics of the medicine that are more likely to be in shortage and whether shortages have changed in frequency over the five-year duration of this study at Mediclinic Parkview (MPAR) Hospital from 2019 to 2023.

An overview of drug shortages in healthcare

According to the United States Food and Drug Administration (US FDA), ‎drug shortage is “a situation in which the total supply of all clinically ‎interchangeable versions of an FDA-regulated drug is inadequate to meet ‎the current or projected demand at the patient level.” The 23rd edition of ‎the World Health Organization’s Model List of Essential Medicines (2023) ‎highlights the critical need for global access to essential drugs, ‎underscoring the importance of managing shortages to ensure equitable ‎healthcare [8].‎

Shortages are due to several causes, such as manufacturing issues in ‎the production facility and workforce, which can be associated with many ‎issues, including quality and regulatory matters [9]. ‎Additionally, economic reasoning, a sudden country withdrawal from the ‎market, or a break period can create a market gap, leading to a drug ‎shortage [2]. When shortages occur, managing the ‎replacement therapy can be complex because drug scarcity can be due to ‎production, trade, regulation, transportation, registration, economic, and ‎external factors that may cause turmoil in the hospital’s ability to deliver ‎quality medical care [10]. The shortage could be caused ‎by an inability to produce, move, sell, or buy sufficient necessary ‎medications [11].

Many reports have indicated drug shortages as a growing concern across several healthcare sectors [10,12]. This phenomenon is not confined to a single geographical region; although drug shortages occur in higher-income countries, they also affect lower-income and developing countries [13]. It is associated with decreased healthcare quality and may lead to medication administration errors, delays in treatment, and adverse patient outcomes [14]. Healthcare professionals worldwide are concerned about the increasing prevalence of drug shortages. Medication shortages worsened during the COVID-19 pandemic and posed a significant threat to the health and safety of patients with COVID-19 and other conditions [15,16].

Pharmaceutical supply chain

The pharmaceutical supply chain is a highly complex system involving multiple stakeholders, from raw materials suppliers to pharmacies and patients. It includes various procedural steps across multiple locations, with critical stages such as manufacturing, marketing authorization, distribution, and patient delivery. This complexity and interdependence across different sectors and countries contribute to potential vulnerabilities that can exacerbate drug shortages [17-20].

According to the systematic review by Sallam [21], enhancing inventory management within ‎hospital pharmacies has been a major area of focus. Dong et al. [22] developed and validated a ‎nomogram to predict hospital drug shortages based on supply chain factors such as procurement ‎volume, therapeutic class, and supplier, offering a tool to optimize hospital drug shortage management.‎

Launching the UAE’s national digital drug tracking system, “Tatmeen” in February 2023, represents a significant advancement in managing drug shortages and ensuring medical supply chain transparency and security. This state-of-the-art platform enables comprehensive tracking and tracing of pharmaceutical products, from origin to expiration, using advanced digital sequencing standards connecting manufacturers, distributors, and healthcare providers, enabling real-time monitoring and timely access to essential medicines while strengthening the medical supply chain [23].

In-depth analysis of global trends in drug shortages

Drug shortages remain a persistent global challenge, with increasing frequency and significant consequences for patients and healthcare systems worldwide. These include limited access to first-line treatments, decreased therapeutic options, and compromised patient care, as noted across various studies [2,24-26]. The economic impact is equally concerning, with rising drug costs and increased healthcare visits attributed to shortages [27-30]. Shortages disproportionately affect low-priced generics, sterile injectables, and nonsolid dosage forms, which are particularly vulnerable due to limited manufacturing incentives [31-33].

Specific regional studies highlight unique challenges and outcomes. In South Korea, better communication between community pharmacies and prescribers improved shortage resolution [34]. Conversely, inefficiencies in communication and a lack of standardized definitions hindered shortage management in South Africa and Denmark [35,36]. Studies in Serbia, the UK, and Germany reported frequent therapeutic substitutions, impacting patient adherence and safety [37-39]. Similarly, shortages of oncology and hematology drugs in Morocco and Pakistan have delayed care and increased patient stress [40-42].

Drug shortages are not limited to high-demand conditions but also affect public health areas like pediatric care and vaccinations. For instance, shortages in pediatric medications and vaccines, such as *Haemophilus influenzae* type B, disrupted immunization schedules and caused spillover effects like extra visits and incomplete catch-up vaccinations [43,44]. Studies in Saudi Arabia revealed frequent shortages in cardiovascular, endocrine, and antineoplastic drugs, with causes ranging from regulatory inefficiencies to poor supply chain management [45-49]. Severe shortages have been documented in Sudan and Colombia, where economic and regulatory challenges compounded by COVID-19 have worsened access to essential medications [50-52].

Globally, the role of pharmacists in managing shortages has been emphasized. Belgian and French studies highlighted the substantial time pharmacists spent searching for alternatives and coordinating solutions [53,54]. Multidisciplinary approaches, as reported in Australian hospitals, have proven effective in mitigating the impacts of shortages [55,56]. Initiatives like tiered allocation systems for critical medications in US tertiary hospitals and district drug shortage monitoring centers in China have demonstrated the value of structured strategies to stabilize supplies [57,58].

Efforts to address shortages extend to improving regulations and procurement processes. Studies in Canada and Jordan pointed to raw material availability, demand surges, and ineffective government regulations as major contributors to shortages [59,60]. In Montenegro, prolonged shortages often affected generic, nonessential drugs, while in Iran, monitoring and policy revisions have been identified as essential strategies to mitigate future challenges [61,62]. Additionally, the global shortage of glucagon-like peptide-1 (GLP-1) receptor agonists highlights the need for regulatory measures to manage demand and protect care continuity for diabetic patients [63].

Research highlights the importance of robust reporting systems, international collaboration, and evidence-based policies to mitigate shortages effectively [64-66]. Advancing therapeutic interchange policies and stewardship programs, improving supplier communication, quality methods, risk management, and adopting innovative technologies can strengthen supply chains and ensure equitable healthcare access worldwide [67-69].

These studies underscored the need for global reforms and targeted strategies to reduce the widespread impact of drug shortages.

Drug shortages in the UAE

For a country like the UAE, with rapid economic growth, developing a world-class medical hub with top-tier hospitals requires strict compliance with international accreditation standards to strengthen medication management, ensure supply chain reliability, and enhance healthcare quality [70]. Also, due to the constant increase in population and the growing number of residents in Dubai, there have been instances of some hospitals with bed occupancy rates reaching almost 100% [71]. In such a demanding healthcare environment with high bed occupancy rates, even a tiny change in the required medication can cause an imbalance that can lead to long hospital pharmacy waiting times, and sometimes, shortages in drugs can cause alterations and cancellations in patients’ treatments [72,73]. This situation causes inconvenience to the patients and healthcare providers [1].

## Materials and methods

Study setting and design

A retrospective descriptive study was performed over 60 months at MPAR ‎Hospital, a 185-bed tertiary care hospital in Dubai, UAE, with more than 46 ‎medical specialties. The hospital belongs to the Mediclinic Middle East (MCME) group. The data were collected from January 2019 to December 2023.

Data source and sample size

Retrospective data were obtained from the MPAR Hospital ‎pharmacy inventory management system and dashboards. The data included records of drug ‎shortages from January 2019 to February 2020 (pre-COVID-19), March 2020 to April 2023 (COVID-‎‎19 Pandemic), and May 2023 to December 2023 (post-COVID-19).

Inclusion and exclusion criteria

The inclusion criteria encompassed all drug shortages reported between 2019 and 2023 at the tertiary care hospital in the UAE, including inpatient, outpatient, and emergency medications. It included drugs that were part of the hospital’s formulary and experienced disruptions in supply or availability, as well as instances where a replacement or therapeutic alternative was required due to the shortage. The exclusion criteria involved medications not included in the hospital’s formulary, those not regularly prescribed during the study period, and drug shortages outside the defined five-year period (2019-2023).

Root cause analysis (RCA)

An Ishikawa diagram, also known as a fishbone diagram, was utilized to pinpoint potential sources and root causes of drug shortages. This method provided a structured approach to identifying and categorizing the contributing factors to shortages across various domains.

Pareto analysis

The Pareto principle, also known as the 80/20 rule, was used to identify the most affected medicine forms and medicine groups by shortages, prioritizing efforts to address these critical areas effectively.

Data analysis

Descriptive statistics were employed to analyze the frequency ‎and trends of drug shortages and their impact on patient care and hospital operations. Data ‎processing and analysis were conducted using Microsoft Excel 2021 and IBM SPSS Statistics for Windows, version 29.0 (IBM Corp., Armonk, NY).

Ethical considerations

The study was exempted from institutional review board (IRB) approval due to its evaluative nature and the type of data involved. The authors reported no conflicts of interest, and the project was conducted without any commercial funding.

## Results

Prevalence of drug shortages

**Figure 1:**
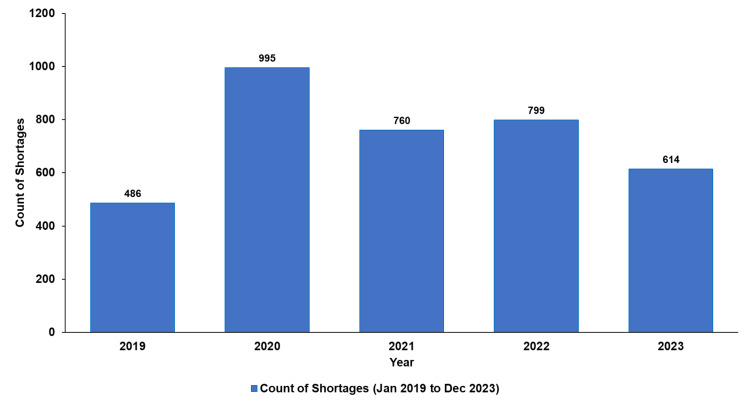
Active drug shortages per year (2019-2023)‎

Figure 1 shows that 3,654 shortages were distributed across the five years of the drug shortages study at MPAR Hospital.

**Figure 2:**
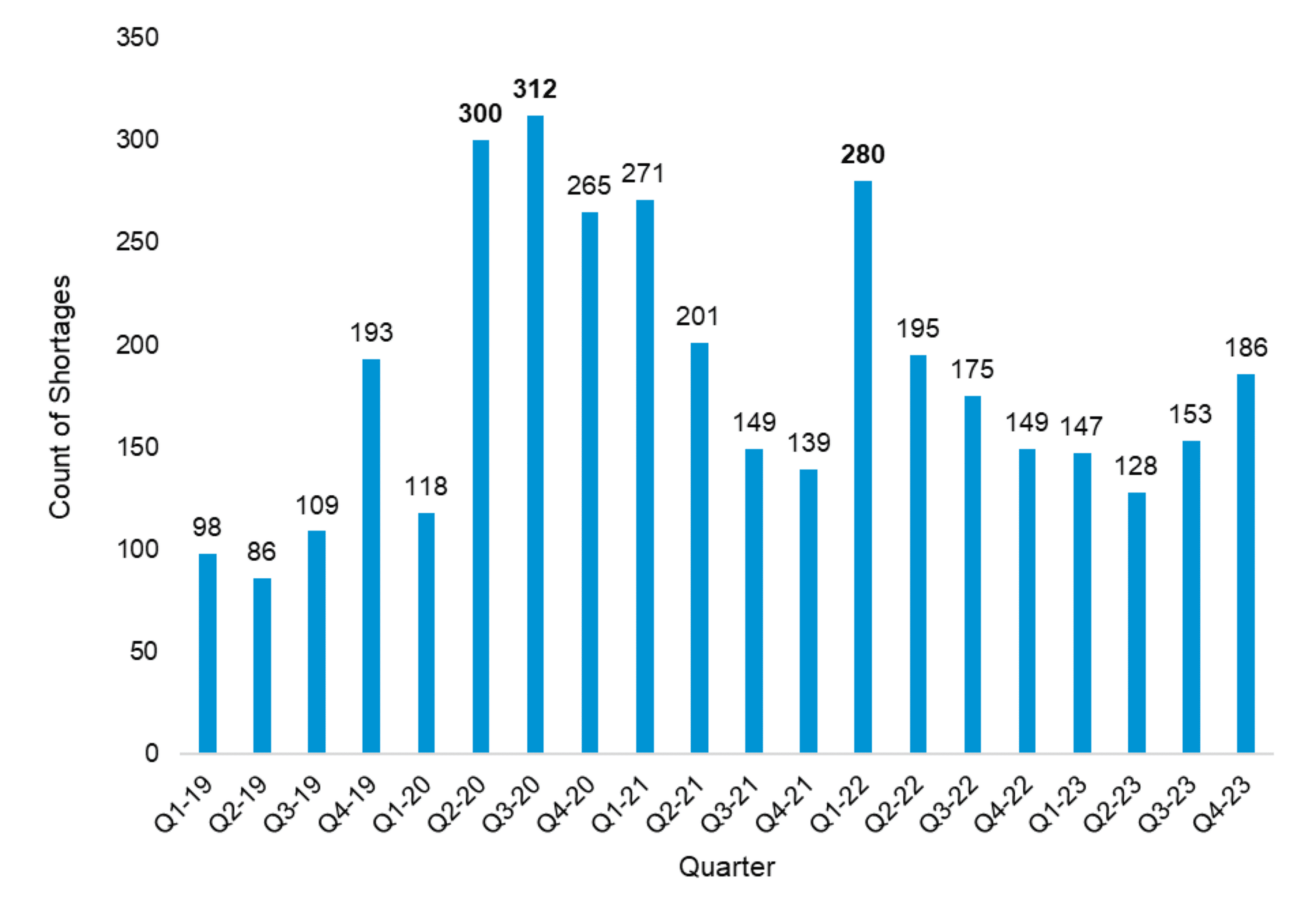
Active drug shortages per quarter (2019-2023)‎

The data illustrate a significant surge in drug shortages, peaking in 2020, with quarterly trends showing fluctuations over the years (Figure 2). This trend highlights the amplified supply chain challenges during the COVID-19 pandemic and the subsequent variations in shortage patterns across different periods.

Pareto analysis of medicine forms and groups affected by shortages

**Figure 3:**
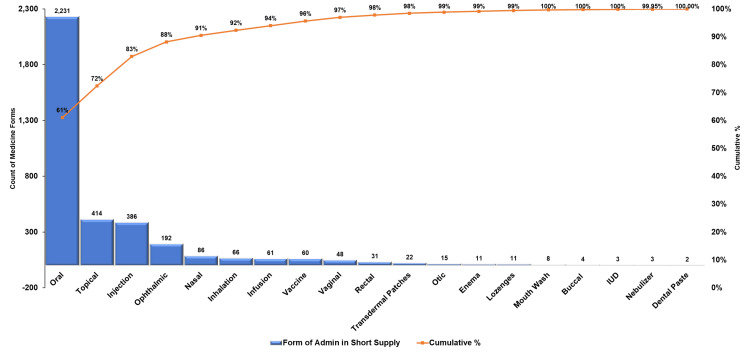
Distribution of medicine forms affected by shortages (2019-2023)‎ IUD, intrauterine device

The Pareto analysis (Figure 3) for medicine forms affected by shortages revealed significant disparities in frequency (n) and percentage contributions among different administration routes. Oral forms accounted for the highest frequency (n = 2231), representing 61% of all shortages, underscoring their critical role in patient care and vulnerability in supply chains. Topical (n = 414, 11%) and injectable forms (n = 386, 10%) followed, cumulatively representing 83% of shortages, indicating that a small number of categories dominated supply issues. Other forms, including ophthalmic (n = 192, 5%) and nasal (n = 86, 2%), contributed marginally to the cumulative percentage, while rare forms, such as transdermal patches and dental pastes, collectively comprised less than 1%. This pattern aligned with the Pareto principle, emphasizing that a targeted focus on the most frequently affected groups, particularly oral, topical, and injectable forms, has lessened the overall impact of medicine shortages.

**Figure 4:**
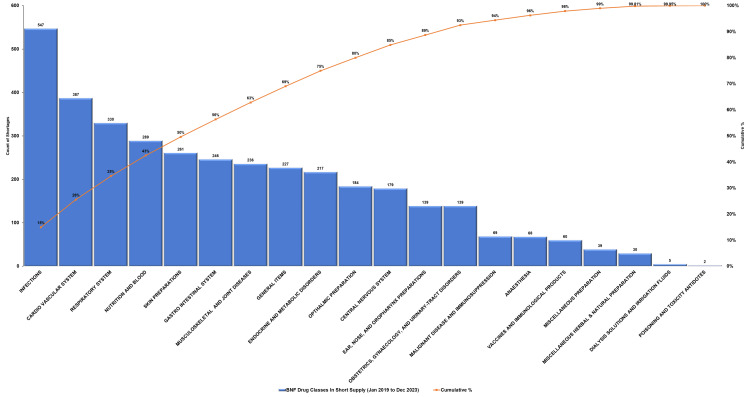
British National Formulary (BNF) drug classes impacted by shortages (2019-2023)‎

Similarly, the Pareto analysis of the therapeutic drug classes impacted by shortages from 2019 to 2023 displayed that a few key categories accounted for the majority of disruptions (Figure 4). Infectious disease drugs experienced the highest number of shortages (n = 547, 15%), followed by cardiovascular system medications (n = 387, 11%) and respiratory system drugs (n = 330, 9%), collectively contributing to 35% of the cumulative shortages. Nutrition and blood, along with skin preparations, accounted for 43% when added. Less frequently affected categories, including dialysis solutions and poisoning antidotes (n = 5 and n = 2, respectively), cumulatively contributed less than 1%. This pattern aligned with the Pareto principle, highlighting the team’s focus on mitigating shortages within the top drug classes.

RCA of drug shortages

**Figure 5:**
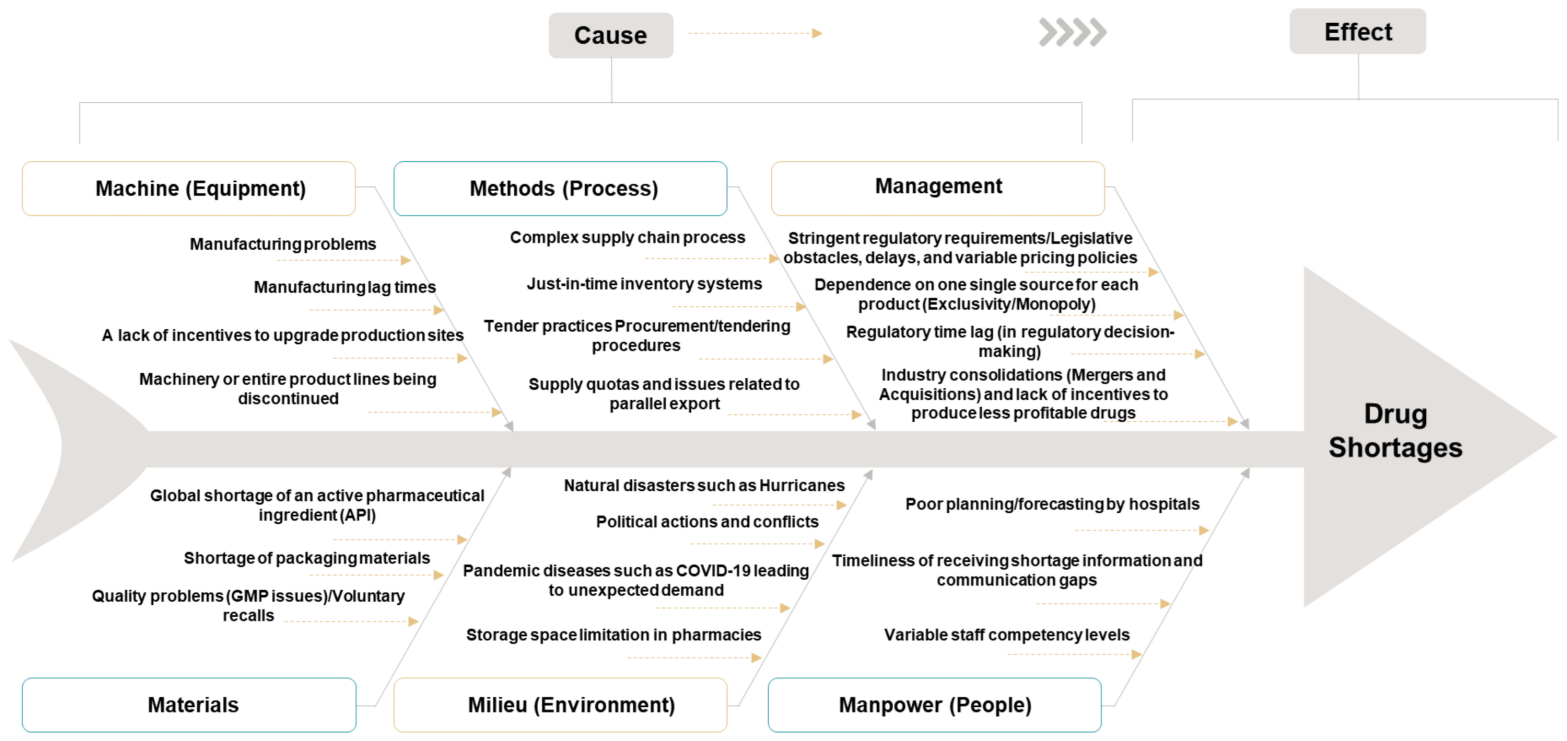
Fishbone diagram describing root causes of drug shortages GMP, good manufacturing practices; API, active pharmaceutical ingredient; COVID-19, coronavirus disease 2019 Image content credit: Dr. Mohammed Sallam

The fishbone diagram (Figure 5) identifies the primary causes of drug shortages across six categories: machine-related issues such as manufacturing lag times, method inefficiencies like complex supply chain processes, material shortages including active pharmaceutical ingredients (APIs), milieu factors such as natural disasters, staffing limitations like staff competency, and management challenges such as regulatory delays, collectively illustrating the multifactorial nature of drug shortages.

Median time spent on drug shortages

Table 1 shows that the median time spent addressing drug shortages peaked at 19.5 days in Q3-2020, with a notable increase in the number of active shortages during this period (312 cases). Despite fluctuations, a downward trend was observed, reaching 8.0 days in Q2-2023 before slightly increasing to 11.6 days in Q4-2023.

**Table 1:** Median time spent on drug shortages Note: The median time spent for each shortage is 90 minutes.

Quarter	Number of active shortages	Median time spent (minutes)	Median time spent (hours)	Median time spent (days)
Q1-19	98	8820.0	147.0	6.1
Q2-19	86	7740.0	129.0	5.4
Q3-19	109	9810.0	163.5	6.8
Q4-19	193	17370.0	289.5	12.1
Q1-20	118	10620.0	177.0	7.4
Q2-20	300	27000.0	450.0	18.8
Q3-20	312	28080.0	468.0	19.5
Q4-20	265	23850.0	397.5	16.6
Q1-21	271	24390.0	406.5	16.9
Q2-21	201	18090.0	301.5	12.6
Q3-21	149	13410.0	223.5	9.3
Q4-21	139	12510.0	208.5	8.7
Q1-22	280	25200.0	420.0	17.5
Q2-22	195	17550.0	292.5	12.2
Q3-22	175	15750.0	262.5	10.9
Q4-22	149	13410.0	223.5	9.3
Q1-23	147	13230.0	220.5	9.2
Q2-23	128	11520.0	192.0	8.0
Q3-23	153	13770.0	229.5	9.6
Q4-23	186	16740.0	279.0	11.6

Source of information about drug shortages

**Figure 6:**
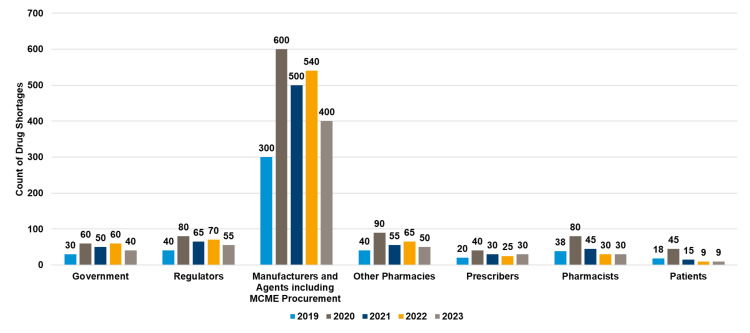
Yearly notifications about drug shortages MCME, Mediclinic Middle East

Manufacturers and agents, including MCME procurement central store, were the most frequent sources of drug shortage notifications, peaking at 600 in 2020 and showing a gradual decline to 400 in 2023, while contributions from other sources such as regulators, pharmacies, and prescribers remained relatively stable over the years (see Figure 6).

Practices for managing prescriptions during drug shortages

**Figure 7:**
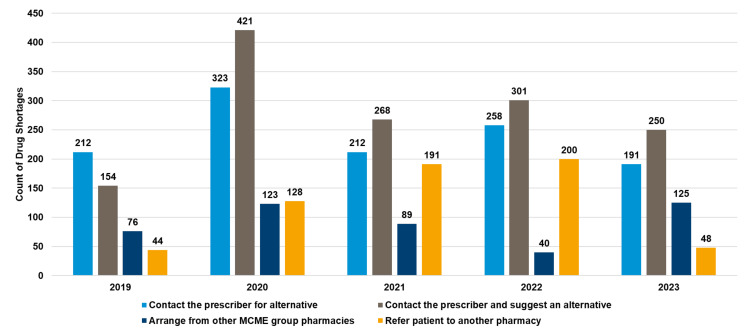
Processes used for managing prescriptions during drug shortages MCME, Mediclinic Middle East

Figure 7 illustrates that the most frequent method for managing prescriptions during drug shortages was contacting the prescriber and suggesting an alternative, peaking at 421 cases in 2020 and arranging alternatives from other MCME group pharmacies, which remained consistently high, reaching 250 in 2023. Referring patients to another pharmacy was the least utilized method, with only 48 cases recorded in 2023.

Impact of medicines shortage

**Figure 8:**
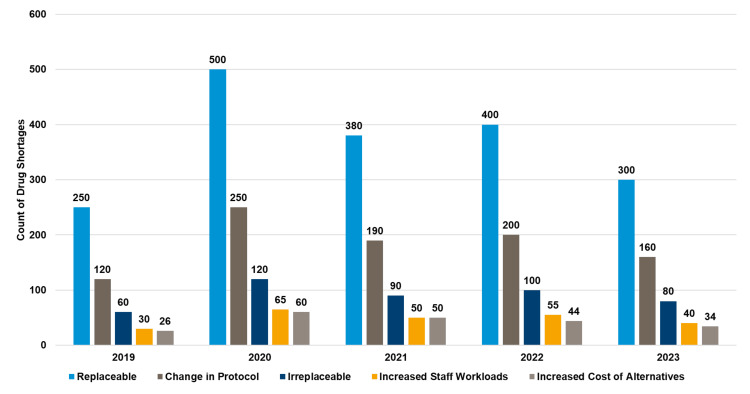
Drug shortages and their impact on the medication use process (2019-2023)‎

In 2020, replaceable drug shortages peaked at 500 cases, while irreparable shortages remained significantly lower across all years, with a slight decline in associated workload and protocol changes by 2023 (see Figure 8).

**Figure 9:**
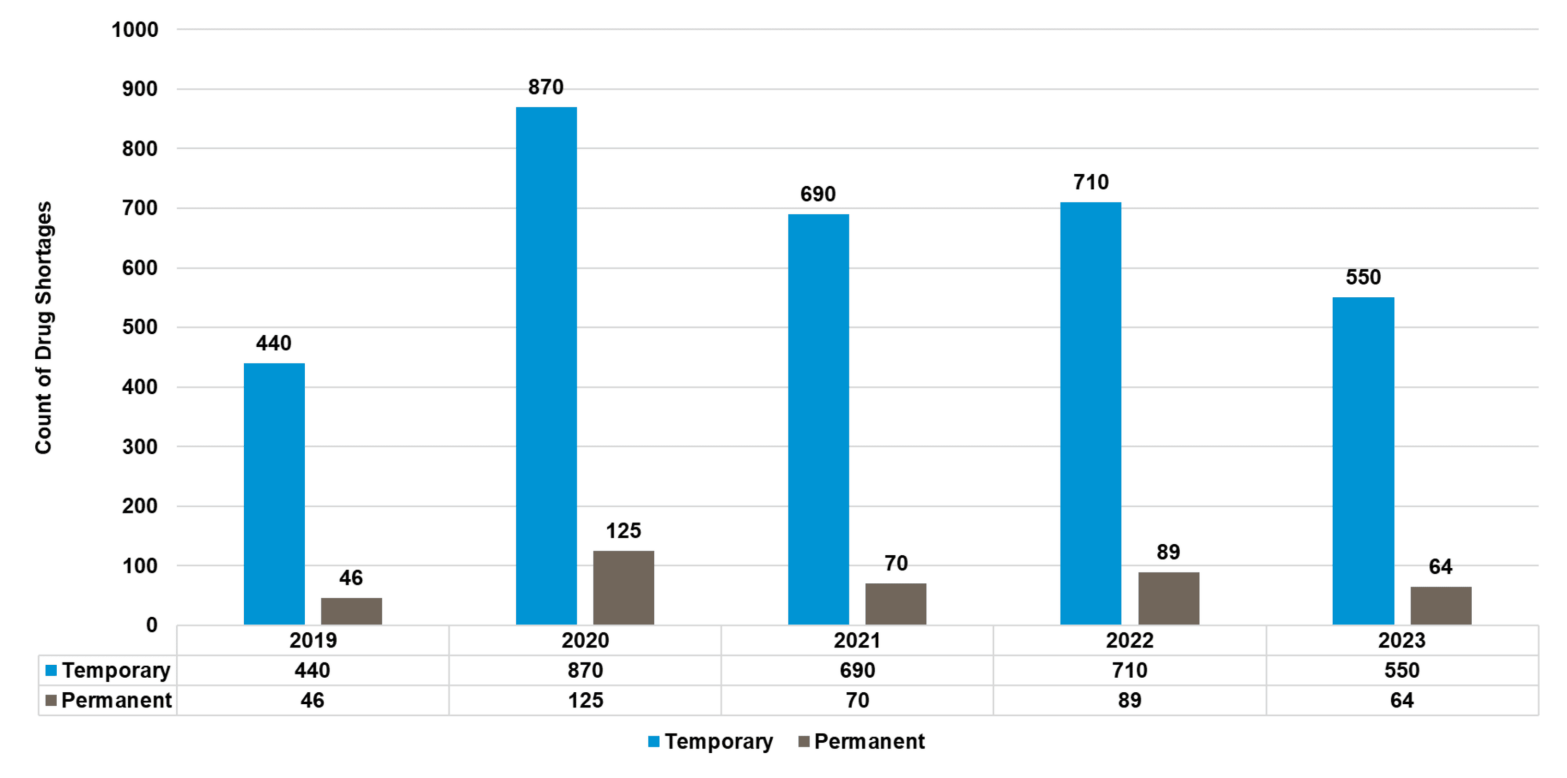
Trends in drug shortages temporary vs. permanent (2019-2023)‎ Drug shortages can manifest as temporary delays in drug availability or permanent drug discontinuations. Temporary drug shortages are situations where the supply of medication is temporarily insufficient to meet the demand or projected demand within a specific timeframe. These shortages are typically resolved once the underlying issues are addressed. Permanent drug shortages, also known as drug discontinuations, occur when a manufacturer permanently stops producing a specific medication. These shortages are more challenging to address and may require finding alternative treatments or sourcing the drug from different manufacturers.

Temporary shortages consistently exceeded permanent ones, peaking at 870 cases in 2020 and decreasing to 550 by 2023 (see Figure 9).

Five-year analysis with comprehensive insights into drug shortages

**Figure 10:**
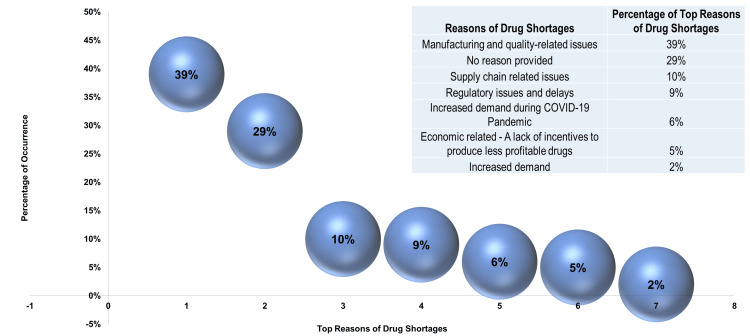
Top reasons driving drug shortages COVID-19, coronavirus disease 2019

From a total of 3,654 reported drug shortages, a random sample of 500 cases (100 for each year) was analyzed. Manufacturing and quality-related issues (39%) emerged as the primary reason for shortages, followed by “no reason provided” (29%) (see Figure 10).

**Figure 11:**
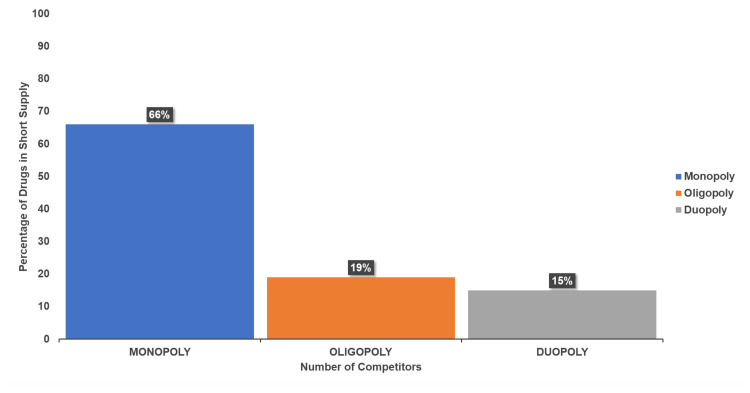
Market competition types contributing to drug shortages Monopoly: A market structure where a single company or entity is the sole provider of a particular product or service, meaning no competitors exist. Oligopoly: A market structure characterized by a small number of firms or competitors that dominate the market. Duopoly: A market structure where only two firms or suppliers control the market for a particular product or service.

The analysis of competition types (Figure 11) revealed that 66% of shortages were linked to monopoly suppliers, while 19% involved oligopolies, and 15% were supplied by two competitors (duopoly). These categories highlighted the influence of competition on drug availability.

**Figure 12:**
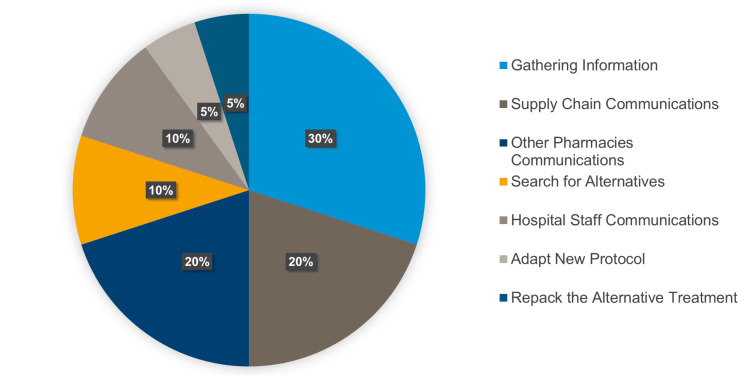
Percentage of task distribution for managing drug shortages

Regarding management efforts, 30% of the time was spent gathering information, with significant contributions from supply chain and pharmacy communications (see Figure 12).

**Figure 13:**
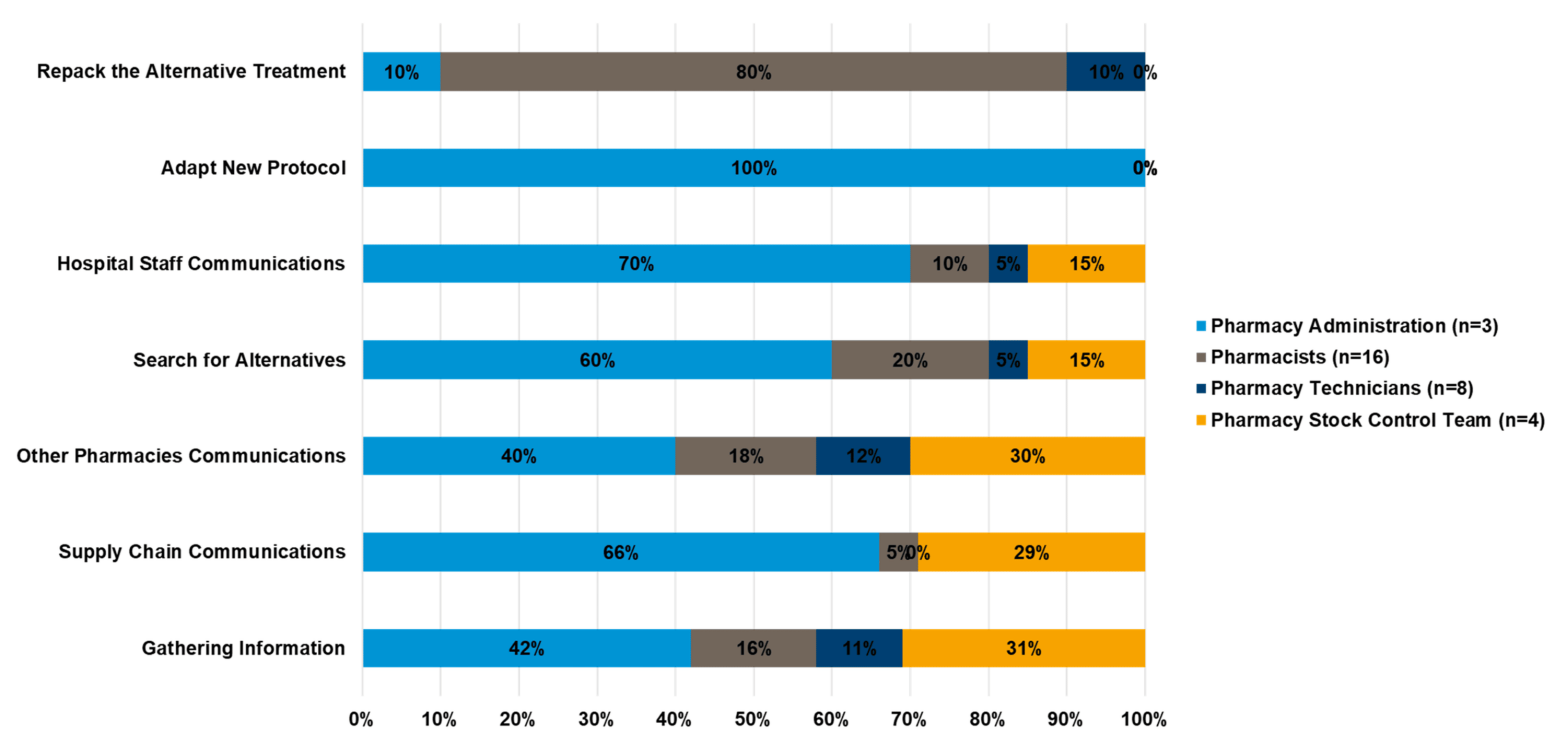
Percentage ‎of time spent by the pharmacy team in managing shortage management

Employees dedicated varying time to managing shortages, with pharmacists focusing on hospital staff communications (70%) and alternative sourcing (60%), while pharmacy technicians led supply chain communications (66%). The stock control team contributed to sourcing alternatives (30%) and inter-pharmacy communication (30%), and administrators focused on adapting protocols (100%) and gathering information (42%) (see Figure 13).

**Figure 14:**
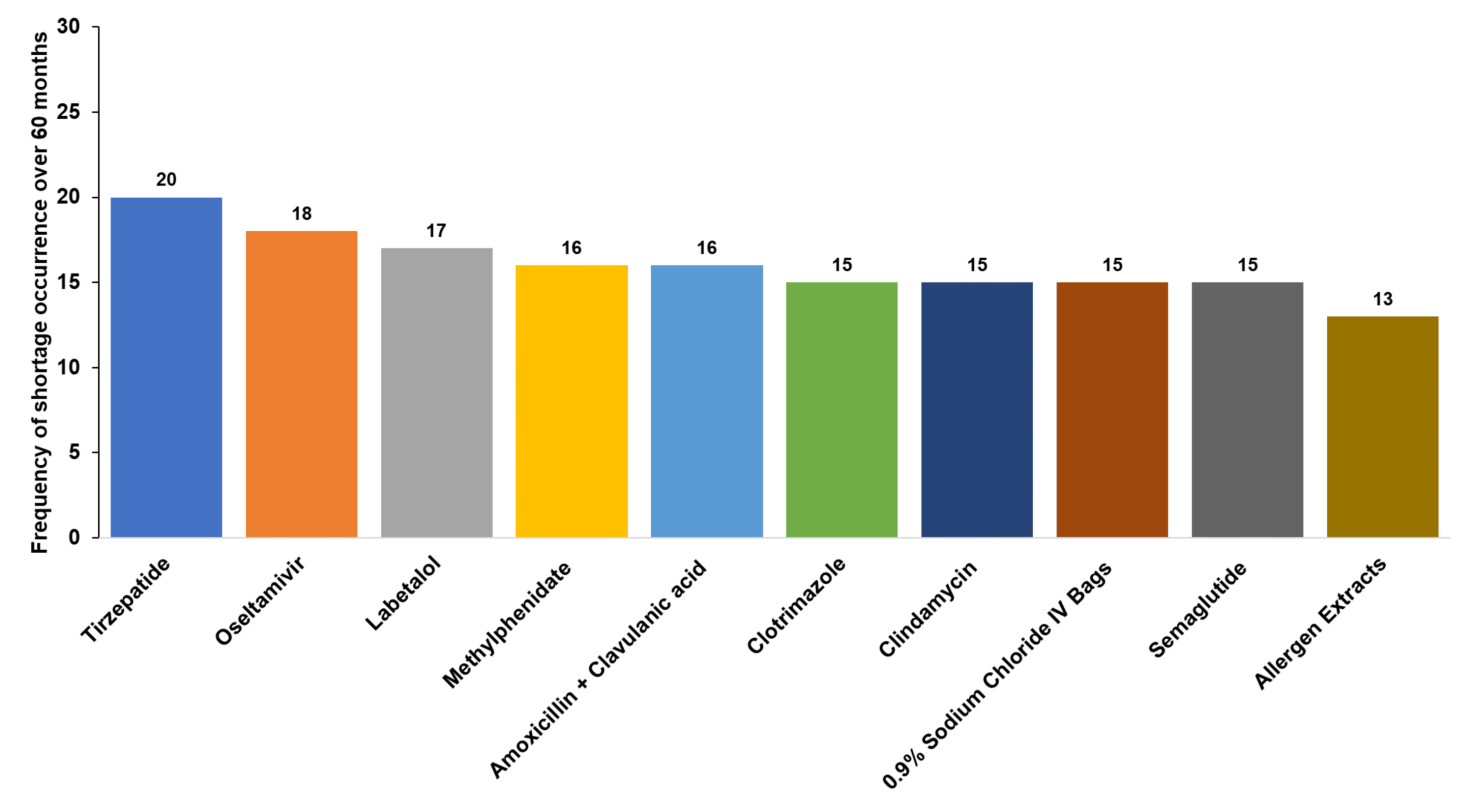
Top 10 drugs with supply disruptions and shortage frequency over 60 months

Figure 14 highlights the top 10 drugs experiencing supply disruptions, with tirzepatide (20 occurrences) and oseltamivir (18 occurrences) leading the list. The frequency of shortages for critical drugs, including amoxicillin-clavulanic acid and 0.9% sodium chloride IV bags, underscores the ongoing challenges in maintaining a consistent supply of essential medications.

**Figure 15:**
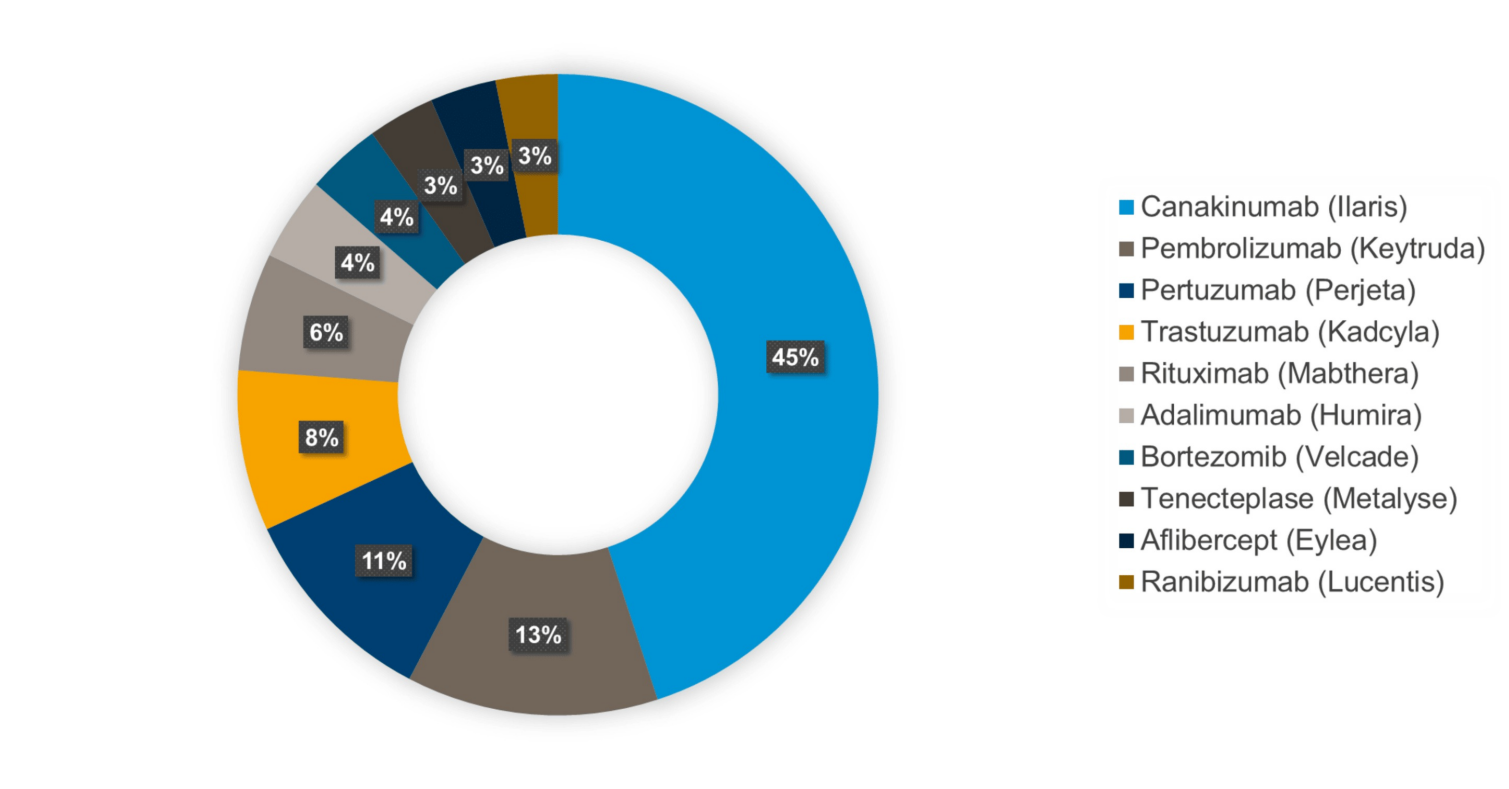
Top 10 costly drug shortages

Figure 15 illustrates the top 10 costly drug shortages, with canakinumab (Ilaris) contributing the largest share at 45%, followed by pembrolizumab (Keytruda) at 13% and pertuzumab (Perjeta) at 11%. High-cost biologics such as trastuzumab (Kadcyla) and rituximab (MabThera) also significantly impacted healthcare budgets. The prominence of expensive oncology and immunology treatments in shortage highlights these disruptions’ financial strain on healthcare systems and the need for strategic planning to secure access to high-cost essential therapies.

## Discussion

The findings from the MPAR Hospital study align with global trends in drug shortages reported across healthcare systems in multiple countries around six continents, including North America [26,27,31,32,43,68,74-79], South America [51,80], United Kingdom [39], Europe [24,25,28,33,35,37,38,53,54,61,67,81-83], Asia [41,42,57,62,84,85], Africa [36,40,50,86,87], the Middle East [11,45-49,60,88-90], and Australia [55,56]. 

The MPAR hospital retrospective analysis revealed distinct trends in drug shortages over the study period, with notable differences between pre- and post-COVID-19 patterns [34,91]. Before the pandemic, drug shortages were primarily linked to manufacturing issues, regulatory delays, and supply chain inefficiencies [91]. However, during the pandemic, the frequency and severity of shortages increased dramatically due to unprecedented challenges such as production halts, transportation delays, export restrictions, and a global surge in demand for essential medications [58,92,93]. These disruptions underscored the fragility of the medication supply chain and highlighted the urgent need for more resilient and adaptable procurement strategies [94]. In the pandemic’s initial phase, hospitals faced immense difficulty procuring critical drugs, resulting in heightened pressure on healthcare providers and the potential compromise of patient care [93,95].

The hospital pharmacy developed a drug shortage dashboard to address these challenges as a pivotal intervention during the pandemic [96]. This tool provided real-time insights into drug availability, enabling decision-makers at the Pharmacy and Therapeutics Committee (PTC) to identify supply gaps, prioritize critical resources, and streamline procurement processes [97]. The dashboard allowed for timely interventions by offering actionable data and proved invaluable in managing escalating shortages. The experience demonstrated the value of data-driven approaches in improving supply chain visibility and responsiveness, particularly during crises, while highlighting the importance of preparedness in healthcare systems [98].

The findings featured significant clinical and ethical implications of drug shortages, particularly during COVID-19 [99]. Shortages of critical medications forced healthcare teams to adapt treatment protocols rapidly, emphasizing the need for proactive planning and enhanced communication among medical staff [13,100]. As key medication experts, pharmacy administrators demonstrated exceptional leadership during this period by implementing structured dynamic frameworks and innovative strategies to mitigate the impact of shortages [101,102]. Their expanded roles in decision-making and resource allocation significantly improved patient outcomes, showcasing the strategic importance of pharmacy leadership in navigating complex healthcare challenges [102].

Tirzepatide shortages exemplified the challenges posed by high-demand medications. The rollout of this innovative therapy was hampered by manufacturing limitations, leaving many patients with delayed or unfilled prescriptions [103]. Oseltamivir shortages, on the other hand, were driven by seasonal spikes in influenza cases, with demand peaking during flu season [104].

This study emphasized the need to strengthen current strategies and explore innovative approaches to mitigate future drug shortages. Key recommendations include diversifying supply chains, fostering international collaboration to reduce dependency on single-source suppliers, and investing in predictive analytics to anticipate and manage potential disruptions. Fostering interdisciplinary collaboration among healthcare providers and integrating robust data tools can further support crisis management and ensure sustained access to essential medications. These findings provide a foundation for improving hospital preparedness and resilience against future supply chain disruptions, safeguarding the quality of patient care, and addressing critical vulnerabilities exposed during the COVID-19 pandemic [52,95,105].

Multidisciplinary approach to managing drug shortages

The study highlights the effectiveness of a multidisciplinary team approach in managing drug shortages [7,55,106]. It emphasizes collaboration among healthcare professionals and the refinement of medication administration guidelines to reduce unjustified and inappropriate medication use while ensuring optimal resource utilization [107]. Key steps taken at MPAR Hospital included optimizing existing stock [101], employing therapeutic alternatives [77,106,108], and maintaining open communication with vendors [19], regulators [109,110], and healthcare organizations [72]. The process began with identifying a drug shortage and operational and therapeutic assessments to evaluate its impact [83]. A thorough shortage impact analysis addressed prescribing, dispensing, and administration challenges alongside financial implications. This structured approach ended in a final plan communicated effectively and implemented seamlessly [13,26]. A well-organized pharmacy, implemented using the 5S Lean methodology: Sort, Set in Order, Shine, Standardize, and Sustain, significantly enhanced medication management by minimizing inventory waste and ensuring efficient storage and retrieval processes, which reduced the likelihood of drug shortages [111].

Study strengths, limitations, and recommendations for future studies

The study demonstrated several strengths, including its comprehensive retrospective analysis over five years, which provided a detailed understanding of drug shortages before, during, and after the COVID-19 pandemic. Using a diverse dataset allowed for identifying key patterns and trends, while the evaluation of real-time interventions, such as the development of a drug shortage dashboard, showcased practical strategies for mitigating shortages. Additionally, the study highlighted the critical role of pharmacists and interdisciplinary collaboration in addressing crises, emphasizing the importance of leadership and coordinated efforts in minimizing impacts on patient care.

However, the study also had limitations. Its focus on a single hospital pharmacy may limit the generalizability of the findings to other healthcare settings. The reliance on internal data introduced the potential for bias. Furthermore, the study did not fully evaluate the long-term outcomes of implemented mitigation strategies or consider broader global factors such as market dynamics and regulatory policies, which could provide additional context to the shortages.

Future studies should include multi-center data and incorporate global supply chain dynamics and regulatory frameworks to improve generalizability and provide deeper insights into mitigation strategies. Research could further investigate the application of time-driven activity-based costing (TDABC) to analyze and optimize resource allocation in managing drug shortages, focusing on identifying cost-effective strategies to improve operational efficiency and patient care outcomes [112-114].‎ Future studies could also explore integrating artificial intelligence (AI), machine learning, and predictive analytics, supported by evidence-based approaches, to anticipate supply chain disruptions better, identify risk factors, and optimize inventory management [115,116]. Research should also focus on strategies to strengthen supply chain resilience through diversified sourcing, international collaboration, proper demand forecasting, and robust risk management frameworks [117]. Additionally, future investigations could assess the impact of structured training programs and greater pharmacist involvement in strategic leadership roles, combined with novel tools, simulation, and drug stewardships, to enhance decision-making and preparedness for addressing future drug shortages [118,119]. As a final recommendation and in light of contemporary trends in the widespread use of social media, platforms like Twitter (X) hold potential for future qualitative research, providing insights into the broader impact of drug shortages on patient populations [120].

## Conclusions

This five-year study offered valuable insights into the frequency, causes, and impact of drug shortages at MPAR Hospital in the UAE, highlighting the complexities of managing shortages in a rapidly growing healthcare system. The hospital’s proactive approach to identifying patterns and evaluating the effectiveness of current mitigation strategies enabled it to anticipate and manage future shortages better, minimizing disruptions to patient care. 

Future efforts should focus on strengthening supply chain resilience and leveraging pharmacy data and machine learning to explore innovative solutions for ensuring the continuous availability of critical medications.
